# Association Between Visual Impairment and Daily Internet Use Among Older Japanese Individuals: Cross-Sectional Questionnaire Study

**DOI:** 10.2196/58729

**Published:** 2024-12-09

**Authors:** Atsuhide Takesue, Yoshimune Hiratsuka, Katsunori Kondo, Jun Aida, Atsushi Nakagomi, Shintaro Nakao

**Affiliations:** 1 Department of Ophthalmology, Juntendo Nerima Hospital, Tokyo, Japan; 2 Department of Ophthalmology, Juntendo University School of Medicine, Tokyo, Japan; 3 Department of Social Preventive Medical Sciences, Center for Preventive Medical Sciences, Chiba University, Chiba, Japan; 4 Department of Gerontological Evaluation, Center for Gerontology and Social Science, National Center for Geriatrics and Gerontology, Obu City, Aichi, Japan; 5 Department of Oral Health Promotion, Graduate School of Medical and Dental Sciences, Tokyo Medical and Dental University, Tokyo, Japan; 6 Division for Regional Community Development, Liaison Center for Innovative Dentistry, Graduate School of Dentistry, Tohoku University, Sendai, Japan

**Keywords:** visual impairment, visually impaired, internet, internet use, internet usage, older adults, digital divide, telemedicine, mobile phone

## Abstract

**Background:**

Older adults might not use computers due to psychological barriers, environmental barriers such as not owning a computer or lack of internet access, and health-related barriers such as difficulties with fine motor skills, low cognitive function, or low vision. Given the health benefits of internet use among older adults, inadequate use of the internet is an urgent public health issue in many countries.

**Objective:**

We aimed to determine whether visual impairment is associated with internet use in a population-based sample of older adults.

**Methods:**

This cross-sectional study sourced data for the year 2016 from the Japan Gerontological Evaluation Study. It included functionally independent community-dwelling individuals aged ≥65 years (N=19,452) in Japan. The respondents reported their visual status by answering the question, “Is your eyesight (without or with usual glasses or corrective lenses) excellent, very good, good, fair, or poor?” We defined “internet user” as a person who uses the internet “almost daily.” We used multivariate logistic regression with multiple imputations to analyze visual status, daily internet use, and any correlations between them.

**Results:**

We observed that 23.6% (4599/19,452) of respondents used the internet almost daily. Respondents with good visual status notably tended to use the internet more frequently than those with poor visual status. Specifically, 13% and 31% of respondents with poor and excellent vision, respectively, used the internet almost daily. In contrast, 66% and 45% of respondents with poor and excellent vision, respectively, did not use the internet. Even after adjusting for several covariates (age, sex, equivalized income, years of education, marital status, depression, history of systemic comorbidities, frequency of meeting friends, and total social participation score), significant associations persisted between visual status and daily internet usage. The odds ratios (ORs) tended to increase as visual status improved (*P* for trend <.001). The adjusted ORs for individuals with excellent and very good visual status who used the internet almost daily were 1.38 (95% CI 1.22-1.56) and 1.25 (95% CI 1.15-1.36), respectively. Conversely, the adjusted OR for those with fair or poor visual status was 0.73 (95% CI 0.62-0.86).

**Conclusions:**

In this study, we reaffirmed what several previous studies have pointed out using a very large dataset; visual impairment negatively impacted daily internet use by older adults. This highlights the need to address visual impairments to promote web use as health care services become more easily accessed on the web.

## Introduction

Visual impairment constitutes a significant component of the global burden of disease. The Global Burden of Disease Study 2015 reported that sensory organ deficits, including vision impairment, ranked second in terms of contributing to years of living with disability worldwide, following lower back and neck pain and preceding depressive disorders [1]. The impact of visual impairment extends to everyday activities, thus imposing a considerable burden on society [2]. A correlation between visual impairment and various health conditions, particularly among older populations, has been extensively documented. Vision loss is associated with several adverse physical outcomes, such as increased risk of motor vehicle accidents [3], falls [4], fractures [5], and difficulties with activities of daily living [6]. Visual impairment impacts the use of community support services among older populations [7]. Therefore, older adults with visual impairments could become isolated without individual support [8-10].

On the other hand, appropriate use of the internet can help increase social contact and therefore decrease the likelihood of social isolation among older adults. Using the internet is associated with physical and cognitive health, health behaviors, and social well-being (more frequent participation in sports groups, meeting friends more frequently, and seeing more friends within a month) [11]. The internet can achieve this by engaging people in activities of interest, gaining social support, connecting to the outside world, and boosting self-confidence [12]. In addition, regular users of the internet have a ~50% lower risk of dementia than nonregular users [13]. Web-based communication with friends or family protects against the probability of developing clinical depression among older Japanese adults [14]. One review emphasized that social media use has several positive effects on the well-being of older adults [15].

However, older adults might not use computers due to psychological barriers, environmental barriers such as not owning a computer or lack of internet access, and health-related barriers such as difficulties with fine motor skills, low cognitive function, or low vision [16]. Whereas previous investigations of older adults with visual impairment are scarce, the following studies offer valuable insights. A small-scale descriptive study [17] and a large-scale case-control study [18] in the United States, along with cross-sectional studies in Finland [19] and the United States [20], reported that visual impairment was a potential obstacle that prevented older adults from using the internet. A cross-sectional study in Singapore [21] used a questionnaire different from previous studies and divided respondents into (1) internet users with health-related difficulties, (2) users without such difficulties, and (3) nonusers for non–health reasons. It found that individuals with visual or hearing impairments, cerebrovascular diseases, and chronic back pain were unlikely to face health-related difficulties with internet use.

Given the health benefits of internet use among older adults, the remarkable development of health-related information technology, and various types of health services delivered mainly via the internet (eHealth) [22], its disparate use is an urgent public health issue in many countries. Japan has one of the most rapidly aging populations in the world. The implications of extending Japanese findings to other countries facing similar demographic shifts in their aging populations have significance for future trajectories. Therefore, we aimed to define whether and how visual impairment and internet usage are associated by analyzing a substantial data set of older Japanese community dwellers aged ≥65 years. Finding that visual impairment hinders daily internet use underscores the importance of addressing this problem and implementing support programs for older adults to promote internet usage.

## Methods

The data for this study were sourced from the Japan Gerontological Evaluation Study (JAGES), which is an ongoing prospective cohort investigation of the social determinants of health among functionally independent individuals aged ≥65 years. The overarching objective of JAGES is to elucidate the social determinants influencing healthy aging. Approximately biennial surveys encompass inquiries about health habits, psychological factors, and an extensive array of social determinants. The surveys are self-administered questionnaires and are distributed via postal mail with the support of local government authorities. This specific study draws upon cross-sectional data derived from the 2016 survey, which was conducted across 39 municipalities between October 2016 and January 2017. The surveyed municipalities spanned the northernmost (Hokkaido) to the southernmost (Kyushu) regions to include urban, suburban, and rural communities in Japan. The selection of these municipalities was not randomized, as the survey was collaboratively undertaken with local municipalities. Questionnaires were sent to all residents aged ≥65 years in municipalities with fewer than 5000 eligible residents, and more populous municipalities were randomly sampled. Among the 279,661 questionnaires distributed, 196,438 were completed, yielding a response rate of 70.2%. The questionnaire comprised core and noncore items, with the former distributed to all targeted populations. Noncore items, comprising 8 modules, were randomly assigned, with respondents receiving 1 module in addition to the core items. The 2016 wave specifically included questions on visual status and internet usage in 1 module of the noncore items, to which 22,295 individuals responded. This focus of the study was directed toward individuals living independently, leading to the exclusion of 2839 participants who either indicated a need for daily care or chose not to respond to the questionnaire. We analyzed data from 19,452 respondents and excluded 4 who did not report their sex. Family members or friends were permitted to help when respondents had difficulty reading or completing the questionnaire.

### Visual Status

Visual status in this study was assessed using a self-administered questionnaire, adapted from the English Longitudinal Study of Aging [23]. The respondents reported their visual status by answering the question, “Is your eyesight (without or with usual glasses or corrective lenses) excellent, very good, good, fair, or poor?” This question is significantly associated with objectively measured visual acuity [24].

### Internet Usage

We quantified the frequency of internet usage based on responses to the standard single-item question, “How often have you used the Internet or e-mail in the past year?” “almost daily,” “2-3 times per week,” “a few times or less per month,” and “none.” We defined an “internet user” as a person who uses the internet “almost daily.” This term encapsulates those for whom daily internet engagement is an essential and inseparable part of their lives. In a similar manner, “internet use” has been defined as daily internet use in other studies [25-27].

### Covariates

We included age and sex as covariates due to their recognized associations with both visual status [28] and internet usage [20,21]. Annual equalized income was also included as a covariate because it correlated with both visual status [29,30] and internet usage [27,31]. The association between educational attainment and both visual status [30] and internet usage [20,27] is well known. Regarding marital status, an association with both visual status [32] and internet usage [20,27] has been previously reported. Given the recognized link between visual status, depression, and internet usage [14,33,34], we included depressive symptoms as covariates. A history of systemic comorbidities is also associated with both visual status [35] and internet usage [19,21,36,37]. Social activities, such as meeting friends or acquaintances and social participation, were included as covariates because they correlated with both visual status [10] and internet usage [11,38]. Age was categorized as 65‐69, 70‐74, 75‐79, 80‐84, and ≥85 years, and equalized household income was classified as low, middle, or high. Educational attainment was grouped as <9, 10‐12, or ≥13 years, and marital status was categorized as married, widowed, separated, or unmarried. Depression was assessed as yes or no, and a history of systemic comorbidities ranged from none to at least 3. The frequency of meeting friends was categorized as <2-3 days per week or ≥2‐3 days per week, and total participation in groups or organizations was scored as 0 (none) to at least 3. We used the Geriatric Depression Scale-15, a 15-item questionnaire with scores ranging from 1 to 15, with higher scores indicating greater depressive symptomatology. Respondents experiencing moderate to severe psychological distress were identified using a cutoff score of 5. Physical health status was assessed by inquiring about a history of systemic comorbidities such as hypertension, stroke, diabetes, blood and immune diseases, musculoskeletal diseases, and eye diseases. Respondents were categorized based on the number of reported diseases: none, 1, 2, or 3 or more diseases (multimorbidities).

To address the potential confounding effects related to social activities, we considered the frequency of meeting friends and acquaintances, categorized as <2‐3 days per week or ≥2‐3 days per week. Social participation was defined as the involvement in any type of social activity during the study period. Respondents were asked how often they participated in volunteer groups, sports groups, hobby groups, senior citizen clubs, neighborhood associations, study or cultural groups, health promotion groups, or activities involving teaching skills or passing on experiences to others. The frequency of participation was assessed as ≥4 times per week, 2‐3 times per week, once a week, 1‐3 times per month, several times per year, or never. We defined “social participation” as participating in a group with a frequency of at least several times per year. We generated a total participation score to assess the intensity of overall social participation. The total number of types of organizations in which each participant participated was tallied, with participation categorized from zero (no participation) to 8 (full participation).

### Statistical Analysis

All variables were analyzed descriptively. We estimated the proportion of visual status (excellent, good, moderate, fair, or poor, respectively) according to age, sex, equivalized income, years of education, marital status, depression, history of systemic comorbidities, frequency of meeting friends, and total participation score. Next, we derived odds ratios (ORs) and corresponding 95% CIs from logistic regression analyses to elucidate the association between internet usage (categorized as “almost daily” vs “<2‐3 times per week”) and visual status. The models used “good” vision as the reference category to estimate the effects of both excellent and impaired vision status. First, we performed a univariate logistic regression analysis, followed by a multiple logistic regression analysis. We adjusted for the following possible confounding factors: age (65‐69, 70‐74, 75‐79, 80‐84, and ≥85 years), sex (men or women), annual equivalized income level (<2 million yen = “low,” 2‐3.99 million yen = “middle,” and 4 million yen or more = “high”), years of education (<9 years, 10-12 years, and ≥13 years), marital status (married, widowed, separated, and unmarried), depression (yes/no), history of systemic comorbidities (none, 1, 2, and ≥3 comorbidities), frequency of meeting friends (<2‐3 days per week or ≥2‐3 days per week), and total participation score (none, 1, 2, and ≥3). We calculated *P* values for trends to determine the linear associations between visual status and internet usage. Missing values were addressed using a multiple imputation approach under the assumption that they were missing at random. Ten imputed data sets were generated using a chained equation, and each data set was analyzed. The results from these data sets were combined using the Rubin method [39]. All the variables in the analyses were used for multiple imputations. The imputation process involved creating regression models for the analyzed variables using chained equations [40]. Logistic, multinomial, and ordinal logistic regressions were applied to the binary, categorical, and ordinal variables, respectively. A history of systemic comorbidities was treated as a binary variable, marital status as a nominal variable, and visual status, frequency of internet usage, years of education, equivalized income, depressive symptoms, frequency of meeting friends, and social participation as ordinal variables in the multiple imputation process. All data were analyzed using Stata 17 (StataCorp; College Station, TX), and significance was set at 5% for the hypothesis tests.

### Ethical Considerations

Ethical considerations were addressed throughout the course of this study. The ethics committee of the Chiba University Faculty of Medicine (#2493) and the National Center for Geriatrics and Gerontology (#992) approved this study. To ensure participant confidentiality, the questionnaires, containing encrypted codes and comprehensive study explanations, were dispatched to individuals via postal mail. This safeguarded anonymity, as the investigators could not identify any individual through the process. The respondents were expressly informed of the voluntary nature of their involvement, and the act of returning the self-administered questionnaire by postal mail was understood as implicit consent.

## Results

**Figure 1.**
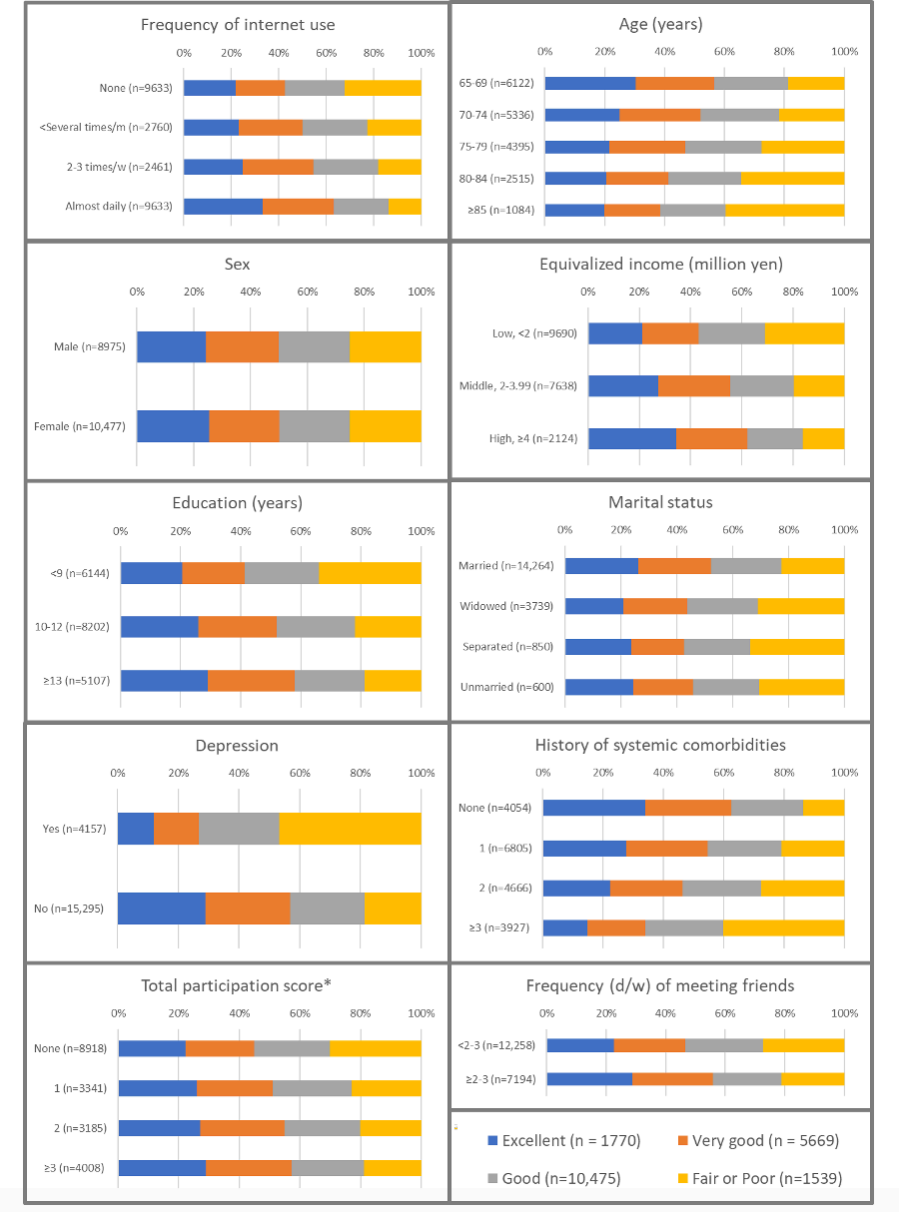
Two-dimensional bar graphs of the descriptive characteristics of study participants by visual status. *Activities with groups or organizations. d: day; m: months; w: week.

The mean age of 19,452 respondents was 73.7 (6.0) (65‒100) years, and 46.1% (8975/19,452) identified as male. Table 1 and Figure 1 provide a comprehensive overview of the fundamental characteristics of individuals categorized by visual status, incorporating multiple imputations. The overall prevalence of internet usage categorized as “almost daily” among the respondents was 23.6% (4599/19,452) (95% CI 23.0-24.28). Respondents with good visual status notably tended to use the internet more frequently than those with poor visual status. Specifically, 13% (201/1539) and 31% (551/1770) of respondents with poor and excellent vision, respectively, used the internet almost daily. In contrast, 66% (1018/1539) and 45% (792/1770) of respondents with poor and excellent vision, respectively, did not use the internet. As age increased, the percentage of older adults who answered that they looked excellent or very good decreased. There was no difference in the visual status between males and females. Respondents with a higher income, more years of education, more opportunities to meet friends, and higher total participation scores tended to answer that they had better eyesight. There were no distinctive trends in marital status. Respondents with depression or a history of systemic comorbidities were more likely to have poor eyesight. For instance, 43% (655/1538) and 11% (194/1770) of the respondents with poor and excellent vision, respectively, had depression. In contrast, 57% (883/1538) and 89% (1576/1770) of respondents with poor and excellent vision, respectively, did not have depression. Of respondents with poor and excellent vision,34% (517/1539) and 12% (220/1770), respectively, had a history of more than 3 systemic comorbidities. In contrast, 11% (173/1539) and 28% (494/1770) of respondents with poor and excellent vision, respectively, did not have a history of systemic comorbidities.

**Figure 2.**
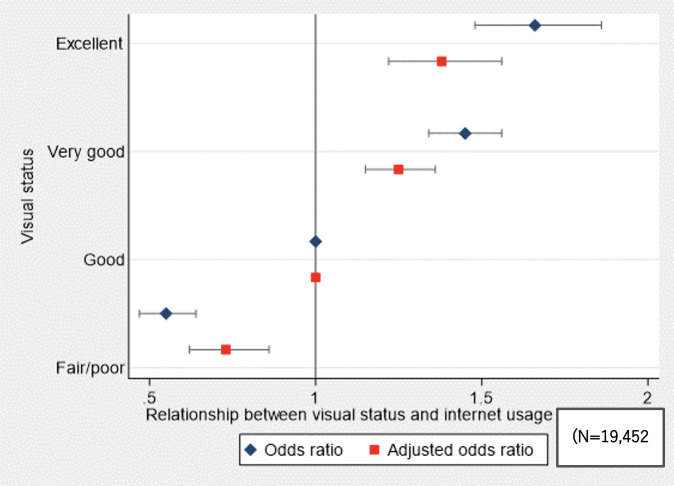
Forest plots of the odds ratios for the relationships between visual status and daily internet usage.

Table 2 summarizes the findings of the univariate and multiple logistic regression analyses, incorporating multiple imputations. The univariate analysis significantly associated visual status with internet usage (*P* for trend <.001). Specifically, the ORs for respondents with excellent, very good, and fair or poor visual status who used the internet “almost daily” were 1.66 (95% CI 1.48-1.86), 1.45 (95% CI 1.34-1.56), and 0.55 (95% CI 0.47-0.64), respectively. Even after adjusting for other covariates, significant associations persisted between visual status and internet use, with a trend of increasing ORs as visual status improved (*P* for trend <.001). Specifically, the adjusted ORs for respondents with excellent, very good, and fair or poor visual status who used the internet “almost daily” were 1.38 (95% CI 1.22-1.56), 1.25 (95% CI 1.15-1.36), and 0.73 (95% CI 0.62-0.86), respectively. Figure 2 shows the forest plots of the ORs for the relationships between visual status and daily internet usage.

**Table 1.** Descriptive characteristics of study participants by visual status (with multiple imputation; N=19,452).

Visual status	Excellent (n=1770)	Very good (n=5669)	Good (n=10,475)	Fair/poor (n=1539)	Total (N=19,452)
**Frequency of Internet use, n (%)**					
None	792 (44.8)	2448 (43.2)	5376 (51.3)	1018 (66.2)	9633 (49.5)
Less than several times per month	222 (12.5)	823 (14.5)	1525 (14.6)	189 (12.3)	2760 (14.2)
2-3 times per week	205 (11.6)	794 (14.0)	1330 (12.7)	131 (8.5)	2461 (12.7)
Almost daily	551 (31.1)	1604 (28.3)	2244 (21.4)	201 (13.0)	4599 (23.6)
**Age (years), n (%)**					
65-69	674 (38.1)	1854 (32.7)	3232 (30.9)	362 (23.6)	6122 (31.5)
70-74	468 (26.5)	1622 (28.6)	2892 (27.6)	354 (23.0)	5336 (27.4)
75-‒79	342 (19.3)	1289 (22.7)	2386 (22.8)	378 (24.6)	4395 (22.6)
80-84	198 (11.2)	638 (11.3)	1389 (13.3)	290 (18.8)	2515 (12.9)
≥85	88 (5.0)	266 (4.7)	576 (5.5)	154 (10.0)	1084 (5.6)
Sex, n (% male)	790 (44.6)	2665 (47.0)	4814 (46.0)	706 (45.9)	8975 (46.1)
**Equivalized income ×10**^**6**^ **yen, n (%)**					
Low	757 (42.8)	2520 (44.5)	5449 (52.0)	963 (62.6)	9690 (49.8)
Middle	739 (41.8)	2436 (43.0)	4000 (38.2)	463 (30.1)	7638 (39.3)
High	274 (15.5)	713 (12.6)	1025 (9.8)	112 (7.3)	2124 (10.9)
**Education (years), n (%)**					
<9	481 (27.2)	1554 (27.4)	3422 (32.7)	687 (44.7)	6144 (31.6)
10-12	749 (42.3)	2412 (42.6)	4491 (42.9)	551 (35.8)	8202 (42.2)
≥13	541 (30.5)	1703 (30.0)	2562 (24.5)	301 (19.6)	5107 (26.3)
**Marital status, n (%)**					
Married	1343 (75.8)	4293 (75.7)	7623 (72.8)	1005 (65.3)	14,264 (73.3)
Widowed	291 (16.5)	1011 (17.8)	2062 (19.7)	374 (24.3)	3739 (19.2)
Separated	80 (4.5)	205 (3.6)	467 (4.5)	98 (6.4)	850 (4.4)
Unmarried	57 (3.2)	159 (2.8)	322 (3.1)	61 (4.0)	600 (3.1)
**Depression, n (%)**					
Yes	194 (11.0)	762 (13.4)	2546 (24.3)	655 (42.6)	4157 (21.4)
No	1576 (89.0)	4907 (86.6)	7929 (75.7)	883 (57.4)	15,295 (78.6)
**History of systemic comorbidities, n (%)**					
None	494 (27.9)	1334 (23.5)	2054 (19.6)	173 (11.2)	4054 (20.8)
1	678 (38.3)	2128 (37.6)	3553 (33.9)	446 (29.0)	6805 (35.0)
2	378 (21.3)	1295 (22.9)	2591 (24.7)	403 (26.2)	4666 (24.0)
≥3	220 (12.4)	912 (16.1)	2278 (21.7)	517 (33.6)	3927 (20.2)
**Frequency (days per week) of meeting friends, n (%)**					
<2‐3	999 (56.5)	3379 (59.6)	6830 (65.2)	1050 (68.2)	12,258 (63.0)
≥2‐3	771 (43.6)	2290 (40.4)	3645 (34.8)	489 (31.8)	7194 (37.0)
**Total participation score** ^**a**^ **, n (%)**					
None	736 (41.6)	2403 (42.4)	4910 (46.9)	868 (56.4)	8918 (45.8)
1	309 (17.5)	958 (16.9)	1837 (17.5)	237 (15.4)	3341 (17.2)
2	308 (17.4)	1003 (17.7)	1675 (16.0)	199 (12.9)	3185 (16.4)
≥3	417 (23.6)	1304 (23.0)	2053 (19.6)	235 (15.3)	4008 (20.6)

a Activities with groups or organizations.

**Table 2.** Odds ratios for the relationships between visual status and daily internet usage (N=19,452) for almost daily internet use.

	Odds ratio	95% CI	*P* value^a^	Adjusted odds ratio	95% CI	*P* value^a^
**Visual status**			<.001			<.001
Excellent	1.66	1.48-1.86		1.38	1.22-1.56	
Very good	1.45	1.34-1.56		1.25	1.15-‒1.36	
Good	1.00	Reference		1.00	Reference	
Fair/poor	0.55	0.47-0.64		0.73	0.62-0.86	

a For trend.

## Discussion

### Overview

To our knowledge, this is the largest cross-sectional study to examine the association between visual status and internet use. The results of our multivariate analysis, adjusted for numerous confounding factors, revealed a significant association between visual impairment and daily internet use among community-dwelling Japanese older adults. Our findings are consistent with those from smaller-scale studies of various designs, indicating that visual impairment serves as a potential barrier to internet usage. A descriptive study in the United States analyzed 45 computer users and reported that barriers to computer use comprised difficulty navigating programs (7%), lack of knowledge (7%), and visual impairment (6%) [17]. A large-scale case-control study in the United States (n=1433) revealed that visually impaired older adults were less likely to use the internet (adjusted OR 0.64, 95% CI 0.49-0.83) [18]. A cross-sectional study in Finland (n=1426) revealed that those older adults with poor near vision (OR 1.90, 95% CI 1.36-2.66) or poor distant vision (OR 1.81, 95% CI 1.21-2.71) had greater odds of not using the internet than their counterparts [19]. The other cross-sectional study in the United States (n=7609) revealed that older adults with vision problems had a lower prevalence ratio (PR) for email or text messaging (PR 0.69, 95% CI 0.58-0.82), health-related internet use (PR 0.44, 95% CI 0.32-0.59), and personal internet use (PR 0.58, 95% CI 0.44-0.77) [20]. In these studies, however, there is no distinction between the internet users with health-related difficulty in its use and the internet users with no health-related difficulty in its use. In a cross-sectional study in Singapore [21], on the other hand, respondents were asked “Do you find it difficult to use internet alone without the assistance of a person or assistive device due to your health or physical state?” Then, they found that individuals with visual or hearing impairments, cerebrovascular diseases, and chronic back pain were unlikely to face health-related difficulties with internet use. Nevertheless, given that both Microsoft [41] and Apple [42] have persistently endeavored to incorporate a range of accessibility features into their products, such as high-contrast features and magnifiers for visually impaired users, it is reasonable to assume that individuals with visual impairments encounter challenges when using the internet.

Whereas numerous people with various types of disabilities face a significant digital divide due to activity limitations, they can equally benefit from the internet if they make full use of it. For instance, a pilot study has reported that the effects of telerehabilitation using the internet on visually impaired individuals were positive [43]. One study of 175 individuals with visual impairment has reported an association between internet use and a sense of well-being [44]. A cross-sectional study targeting Chinese individuals aged ≥45 years (n=17,433) revealed a positive association between visual impairment and depression, while internet use and social participation were important mediators that mitigated the effects of visual impairment on depression. In the mediation analysis, the internet use pathway contributed to 37.72% of the total effect, and the social participation pathway accounted for 52.69% [34]. As the concept of the internet of things (IoT) becomes more widespread, older adults with visual impairment can become beneficiaries [45]. For example, a smart IoT-based mobile sensor unit attached to a cane was developed for visually impaired people. The IoT-based mobile sensors unit consists of a microcomputer, GPS sensor, accelerometer, cameras, laser or ultrasonic sensors, and a digital motion processor [45,46]. A scoping review highlighted that certain smartphone features, such as zoom and magnification, are helpful for those with low vision, but text input and output, and commands using speech (eg, Siri), are useful for those who are blind [47].

One review discussed factors influencing eHealth’s success and failure, including privacy, security, patient empowerment, quality of health care, workflow, and costs [48]. Another review highlighted barriers to telemedicine adoption, such as technical challenges, resistance to change, costs, patient age, reimbursement, and education level [49]. However, practitioners should recognize that unequal internet access due to visual impairment among older adults could widen the digital divide between those with and with no normal vision. Developers should prioritize accessibility in their web designs to improve the web-based experience for visually impaired older adults. Ophthalmology professionals should raise the bottom of older adults’ visual functions as a whole to promote internet use. Restoring visual function is often difficult in older adults with central visual field damage caused by age-related macular degeneration, chorioretinal atrophy, or severe glaucoma. However, the prevalence of diabetic retinopathy and cataracts is high in Japan, and at least 30% of these incidents are preventable and treatable [50]. In Japan, the epidemiology of refractive errors among older adults varies significantly between urban and rural islands. The prevalence of myopia was considerably lower on the island (18.6% vs 32.4% in urban areas), whereas that of hyperopia was higher (34.1% vs 27.9%) [51]. Our survey of uncorrected refractive errors among older adults in rural mountainous areas found prevalences of 11.96% (353/2952) for those aged 70‐79 years and 22.39% (661/2952) for those aged ≥80 years [52]. Therefore, expanding cataract surgery, providing appropriate spectacles, and prescribing low-vision aids can address many visual problems in older adults. Improving the visual function can enhance internet use.

### Strengths and Limitations

This study has strengths. We analyzed the largest population-based data set ever. The statistical power allowed us to adjust for covariates including socioeconomic status, social activities, and psychological and physical health status. We analyzed data from areas of Japan where the population is aging and the internet infrastructure is progressing. Previous studies have included the descriptions “internet use,” “past and/or present use of the internet,” “had used the internet in the past 12 months,” or “used the internet in the last month,” [17-21]. In contrast, “internet use” herein refers to daily internet use based on other studies [25-27]. Evaluating the impact of daily internet usage in contemporary life seems warranted, considering its increasing importance.

We cannot completely exclude the possibility of reverse causation owing to the cross-sectional nature of the data. While visual status affects internet use, the opposite might be true, because the internet can increase healthy motivation among older adults by providing daily communication with people from the same generation and access to health-related information. For example, those with smaller support networks are less likely to receive cataract surgery [53].

According to the Communications Usage Trend Survey published by the Japanese Ministry of Internal Affairs and Communication, possession rates of smartphones by persons aged in their 60s were 45.1% in 2017 and 91.5% in 2022 [54]. Our data were collected in 2016, which may have affected the relevance of the results. Given the rapid evolution of the internet industry, advancements in technology could have led to improved accessibility and different patterns of internet use among people with impaired vision. As time progresses, it becomes increasingly common for older individuals who are already using the internet to develop visual impairments. Future studies should explore these evolving trends to provide a more accurate understanding of the relationship between visual impairment and internet use.

Due to the limitations of our data, we could not assess the duration of internet use. Our study aimed to explore the relationship between visual impairment and the frequency of daily internet use. Future studies should investigate whether visual impairment affects the intensity of internet use from both low (low usage) and high (addiction) perspectives.

The data from the JAGES study relied on self-reporting, which introduces the potential for recall and social desirability bias. Self-reported vision in this context reflects the presented vision rather than the best-corrected vision. Self-reported information slightly overidentifies visual impairment compared with measured visual acuity [55], and the concordance between these 2 measures varies across sociodemographic groups [56]. Despite this limitation, a recent study using big data from the United States found no differences in the general direction of associations between the social determinants of health and vision loss, regardless of whether clinically evaluated or self-reported vision measures were used [57]. The multidimensional nature of self-reported vision is noteworthy, as it encompasses various aspects that directly influence the lives of older individuals, particularly under challenging conditions such as low and fluctuating light levels, glare, and low contrast [58]. Consequently, our data are likely intricately linked to the vision-related quality of life of the respondents.

### Conclusions

Visual impairment negatively affects daily internet use among Japanese older adults. To enhance internet usage among older individuals, addressing visual impairment must be a key consideration.

## Abbreviations

IoT: internet of things
JAGES: Japan Gerontological Evaluation Study
OR: odds ratio
PR: prevalence ratio
